# Interfacial Strengthening and Self-Monitoring in Carbon Fiber-Reinforced Composites via Carbon Nanotube-Based Damage Sensors

**DOI:** 10.3390/nano12213717

**Published:** 2022-10-22

**Authors:** Wenlong Hu, Zijie Sun, Lulu Yang, Chaojie Hu, Shuzheng Zhang, Fangxin Wang, Bin Yang, Yu Cang

**Affiliations:** 1 School of Aerospace Engineering and Applied Mechanics, Tongji University, Shanghai 200092, China; 2 Beijing Spacecrafts, China Academy of Space Technology, Beijing 100094, China; 3 College of Civil Science and Engineering, Yangzhou University, Yangzhou 225127, China

**Keywords:** MWCNTs, carbon fiber, interfacial properties, sensors

## Abstract

Carbon fiber-reinforced polymers are important constituents of aerospace materials. However, due to the inert surface of CFs, their interfacial property is relatively weak, which severely hinders their practical applications. Here, we deposited multi-walled carbon nanotubes (MWCNTs) along with a coupling agent on the surface of carbon fiber to improve the interfacial properties of the carbon fiber/resin. Via a simple dip-coating method, the MWCNTs were uniformly distributed on the CF surface with the assistance of the pre-coated coupling agent. The interfacial shear strength between the fiber and the matrix was significant enhanceed when the CF was loaded with the coupling agent and the MWCNTs. In addition, the MWCNTs were used as sensors to in-situ monitor the interfacial state in order to elucidate the interfacial strengthening mechanism. It revealed that the collaborative contribution of the coupling agent and the MWCNTs in the interphase region is the key to the high interfacial strength.

## 1. Introduction

Carbon fiber-reinforced polymer composites (CFRPs) are well known for their outstanding mechanical properties at low densities, which makes them one of the most popular materials in the aerospace and automotive industries [1,2,3]. CFRPs inherit the merits of both carbon fibers and polymer matrices and, hence, manifest exceptionally high strength-to-weight and stiffness-to-weight ratios, which enable them to substitute metal-based composites in high-performance structural applications [4,5,6]. Given their practical purposes, improving the mechanical performances of CFRPs has attracted a lot of research interest since the concept of CFRPs was proposed [7,8]. Enhancing the mechanical properties of each constituent and optimizing their balance have been carefully considered [9,10,11]. Besides that, the interfacial properties between the carbon fiber and the polymer matrix play an important role, the control of which is of great importance to ensure efficient load transfer from the matrix to the reinforced counterparts.

From the weak polymer matrix to the strong reinforcement of carbon fibers, there is an interfacial layer (also called “interphase”) with distinct physical and chemical properties that displays good affinity with both constituents and, hence, guarantees a favorable load transfer capability. However, the surface of pristine carbon fiber is smooth and adheres poorly to the polymer matrix, leading to cracks easily propagating along the interfacial layer [12,13]. This inherent disadvantage limits the practical applications of CFRPs; therefore, there are numerous research studies on enhancing the interfacial properties by forming either higher amounts of physical interlocking or higher amounts of chemical bonding between carbon fibers and matrix [14,15,16]. Incorporating nanomaterials onto the surface of carbon fibers is a hot research topic on strengthening the interfacial adhesion and the mechanical properties of CFRPs [17,18,19]. In light of the excellent mechanical properties, emerging nanomaterials, such as carbon nanotubes (CNTs) [20,21] and graphene and their derivatives [22,23], acting as reinforcement have been extensively studied. CNTs, reported by Ijima in 1991 [24], are ideal candidates for reinforcements, given their low density (~1 g/cm^3^), large aspect ratio (>1000), and exceptional mechanical properties (Young’s modulus: 1.2 TPa) [25].

Modifying the surface with CNTs could be achieved by different technologies, such as replacing the sizing agent pre-coated on the surface of carbon fiber [26,27,28], electrophoretic deposition [29,30], and chemical vapor deposition [31,32,33]. The conventional method is the sizing technology, which replaces the original sizing agent with a new agent that has a better affinity with CNTs. Although this approach can distribute the CNTs uniformly on the fiber surface, a certain degree of degradation of fibers is inevitable during the desizing treatment and hence impact the mechanical performance of fibers [34,35]. Alternately, physical approaches, such as spraying and electrophoretic deposition [36,37], have been recommended; however, a pre-treatment of the fiber surface is required, which turns out to be complicated and time-consuming [38,39]. Therefore, an approach that is relatively fast and does not sacrifice the fiber’s mechanical properties is important for practical applications. Moreover, the interfacial properties are affected by complex factors, including the microstructure and morphology of the CNTs, the chemical/physical bonding between the CNTs and the sizing agent or the matrix, and the serving environments. Therefore, a direct and quantitative measurement of the interface is necessary, which is, however, still limited.

To enhance and characterize the interfacial properties of the CFRPs, in this work, we explored a simple and timesaving approach of dispersing multi-walled carbon nanotubes (MWCNTs) uniformly on the surface of the carbon fiber by using a commercial coupling agent. We investigated the optimizing combination of the coupling agent and the MWCNTs, and measured the corresponding interfacial properties by performing single-fiber pull-out tests. By characterizing the morphology of the pull-out fibers and monitoring the interfacial properties via the electrical resistance of the CNTs, the interfacial strengthening mechanism is elucidated. This method shows great potential in modifying the interfacial properties of CFRPs.

## 2. Materials and Methods

### 2.1. Materials

Carbon fiber yarns (PAN-based, with a diameter of 1 mm) were supplied by Guangwei Composite Material Co., Ltd., Weihai, China. The carbon fiber yarns were desized using an alcohol lamp (with a temperature of 650 °C and a heating duration of 3 min). 3-glycidyl ether oxy-propyl trimethoxy silane (KH560) (98%) was provided by Chuangshi Chemical Additives Co., Ltd., Nanjing, China. Carboxyl functionalized multi-walled carbon nanotubes (MWCNTs) (with an outer diameter between 30 and 80 μm, length < 10 μm, and specific surface area > 60 m^2^/g) (>98%) were obtained from the Chinese Academy of Sciences Chengdu Organic Chemistry Co., Ltd., Chengdu, China. Sodium dodecyl sulfate (SDS) was supplied by Kunxiang Chemical Technology Co., Ltd., Ningbo, China. Ethyl alcohol was purchased from Feilong Chemical Co., Ltd., Suzhou, China.

### 2.2. Surface Modification on the Surface of Carbon Fiber

**Figure 1:**
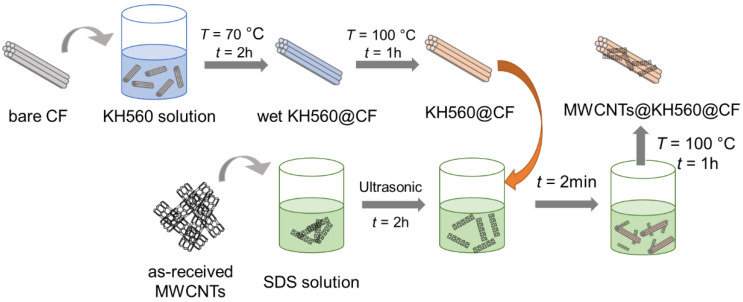
Schematics of the surface modification process on the carbon fiber yarn.

The carbon fiber’s (CF) surfaces were modified with multi-walled carbon nanotubes following the procedure schematically shown in Figure 1. First, the silane coupling agent (KH560), as a linker between the CF and the MWCNTs and/or the CF and the polymer matrix, was coated on the surface of the CFs. The KH560, with a mass of 1–10 g, was dissolved in 85.5 mL of anhydrous ethanol containing 4.5 mL of distilled water, and the mixture was stirred using ultrasonic waves for 30 min. The pH value of the mixture was adjusted to 5 using acetic acid to improve the hydrolysis efficiency of the KH560. Then, the carbon fiber yarns, with an average length of 10 cm, were dispersed in the solution at 70 °C for 2 h, giving rise to a thin layer of KH560 coated on the CF surfaces. The KH560-coated CFs, named as n-K@CFs, were dried at *T* = 100 °C for 1 h in a convection oven, where *n* (= 1, 3, 5, and 10) and K denotes the weight fraction of the KH560 (K) in *n* percent. In the second step, 250 mg of MWCNTs and 75 mg of surfactant (SDS) were dispersed in 40 mL of distilled water and then vibrated using ultrasonic waves for 2 h to disperse the MWCNTs. The dried n-K@CFs, prepared in the first step, were immersed in the MWCNT dispersion for 2 min to adsorb the MWCNTs onto their surfaces. The content of MWCNTs that were coated on the CF surface was controlled by the weight fraction of the KH560. The n-K@CFs with MWCNTs, denoted as M-n-K@CFs, were dried at *T* = 100 °C for 1 h to obtain MWCNT-modified CFs. The CF surface’s morphology before and after modification was observed using a scanning electron microscope (SEM, JSM6010, JEOL, Tokyo, Japan).

### 2.3. Single-Fiber Pull-Out Test

**Figure 2:**
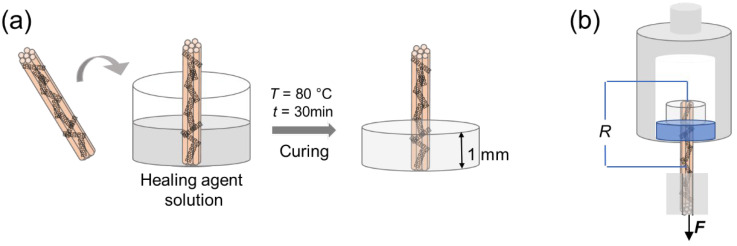
Schematics of (**a**) sample fabrication and (**b**) single-fiber pull-out test.

We performed a single-fiber pull-out test to explore the interfacial properties of the MWCNT-modified carbon fibers (M-n-K@CFs). As a typical specimen for this testing, parts of the M-n-K@CF yarn are embedded in the polymer matrix as schematically shown in Figure 2a. Experimentally, epoxy resin (TECHSTORM 481) was filled in a vessel with a diameter of 10 mm, carrying the vertically positioned M-n-K@CF yarn in its center, and then solidified at *T* = 80 °C for 30 min. The length of the embedded fiber yarn was set to 1 mm. As control samples, the CF and n-K@CF that were partially embedded in the resin were prepared in the same fashion. Pull-out measurements were performed using a universal testing machine (Instron 34TM-30, Instron Corporation, Boston, MA, USA), as shown in Figure 2b. The fiber yarn’s end with the matrix was restrained by a designed fixture with a hole (diameter of 4 mm), as shown in Figure 2b, while the free end of the fiber was pulled out at a constant speed of 2 mm/min. In order to avoid stress concentration, two gaskets were used at the interface between the fiber and the grips. The test continued until the fiber yarn was completely pulled out of the matrix when the pull-out force F was zero. The pull-out load against displacement was recorded using a computer-controlled plotter. The interfacial shear strength (IFSS), *τ*, was estimated according to the following equation:

*τ* = *F*_max_/π*DL* (1)

where *F*_max_ is the maximum debonding force (N), and *D* and *L* are the diameter and length of the CF embedded in the matrix, respectively, which were determined by a digital microscope (Boshile-1000, Boshile Ltd., Jinhua, China). Each test was repeated five times. The CF surface’s morphology before and after modification was observed using a scanning electron microscope (SEM, JSM6010, JEOL, Ltd., Tokyo, Japan).

### 2.4. Self-Monitoring Process

MWCNTs could present multi-functionalities, given their high electrical conductivity and thermal conductivity. In this work, we employed MWCNTs as sensors for in-situ monitoring of the interfacial properties by recording their electrical resistance changes, as shown in Figure 2b. Two ends of the CFs were connected by a wire, between which was the resin block. In the pull-out test, the real-time resistance changes of the M-n-K@CF’s interfacial sensors were continuously recorded using a Keithley 2700 programmable electrometer.

## 3. Results

### 3.1. Surface Morphology and Electrical Resistivity of Modified CFs

**Figure 3:**
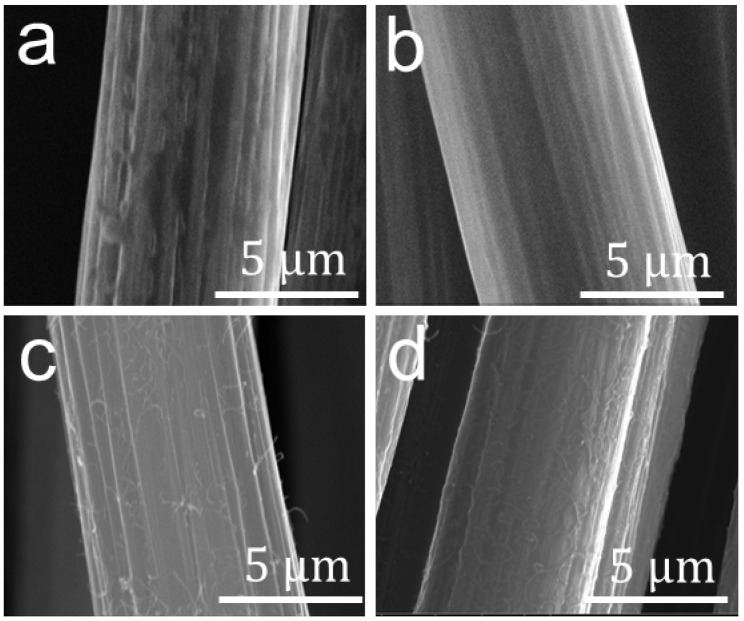
SEM images of (**a**) bare CF, (**b**) 10%-K@CF, (**c**) M-5%-K@CF, and (**d**) M-10%-K@CF.

The surface of the CFs was modified with the MWCNTs following the procedure illustrated in Figure 1. The evolution of the surface morphology of the CFs was characterized using SEM, as shown in Figure 3. The receiving CF underwent a desizing treatment at *T* = 650 °C for 3 min, where the commercial sizing agent was decomposed, but CF degradation did not occur, as proven in our previous study [40]. The obtained bare CF in Figure 3a exhibits several longitudinal grooves, which come from its spinning process. After being coated with KH560, the surface grooves on the fibers, such as the 10%-K@CF in Figure 3b, become distinctly shallower, in comparison to the bare CF in Figure 3a, because the additional silane agent (KH560) layer fills in and, hence, smooths the grooves. In fact, the surface roughness of the KH560-modifed CF decreases with an increasing concentration of the KH560, as shown in Figure S1, which directly proves the effectiveness of the modification approach. As a linker between the CFs and the polymer matrix, the KH560 layer facilitates the loading of the MWCNTs onto the surface of the CFs. Figure 3c and Figure S1 show the surface of the MWCNT-modified CFs with and without a KH560 layer, respectively. With the KH560 layer, CFs apparently absorbs more MWCNTs on its surface. The amount of MWCNTs increases with an increase in the KH560 weight content. For the M-10%-K@CF with the largest KH560 content, the MWNCTs are agglomerated, as shown in Figure 3d, while the homogenous distribution of MWCNTs with a diameter of 40–80 nm (Figure S3) is observed for other samples with lower KH560 content.

**Figure 4:**
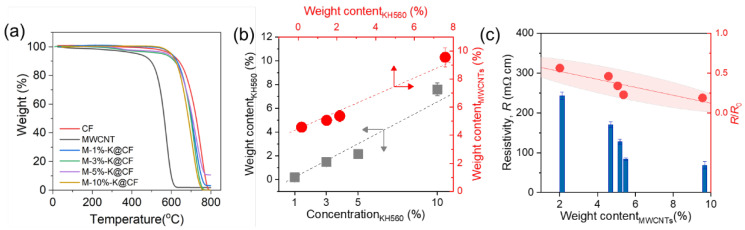
(**a**) Weight fraction as a function of temperature for CFs with and without MWCNTs, from which the weight fraction of KH560 and MWCNTs in (**b**) is estimated. The electrical resistivity R (blue columns) as a function of MWCNTs is shown in (**c**). The arrows indicate the data belong to the corresponding axis and the dotted lines are guides to the eye. The red area in (**c**) denotes the 95% confident ellipse (mean) of R/R_0_ (red square).

To elucidate the relationship between the KH560 and the MWCNTs, we quantified the weight content of the KH560 and the MWCNTs by performing thermogravimetric analysis (TGA) tests, as shown in Figure 4a and Figure S2. Figure 4b displays the relationships between the weight content of the KH560 and its concentration and the weight content of the MWCNTs. For the former, the weight content of the KH560 coated on the CF surface increases linearly with its solution concentration, as expected. As its concentration increases from 1% to 10%, the weight content of the KH560 increases from 0.2% to 7.6%. The increase in the KH560, in turn, leads to an enhancement in the weight content of the MWCNTs, which increases from 4.6% to 9.5%. Therefore, experimentally, we could control the weight content of the MWCNTs by adjusting the concentration of the KH560 during the fabrication process. It should be noted that the initial weight loss in Figure 4a results from the decomposition of the KH560 (Figure S2).

To further prove that the weight content of the MWCNTs increases with the KH560, we measured the electrical resistivity R of the M-n-K@CFs, as shown in Figure 4c. The electrical resistivity (R), as measured using a probe resistivity instrument (M-6, Huangkun, Ltd., Suzhou, China), monotonically decreases with increasing amounts of MWCNTs, which is expected given the high electrical conductivity of the MWCNTs. The normalized R/R_0_ decreases from 50% to 10% as the weight content of the MWCNTs increases from 1% to 9%, where R_0_ is the electrical resistivity of the corresponding n-K@CF without MWNCTs. The sensitivity of R/R_o_ to the MWCNTs’ properties (e.g., content and distribution) could be utilized to monitor the interfacial properties in the region where the MWCNTs are located.

### 3.2. Interfacial Properties of MWCNT-Modified CFs

**Figure 5:**
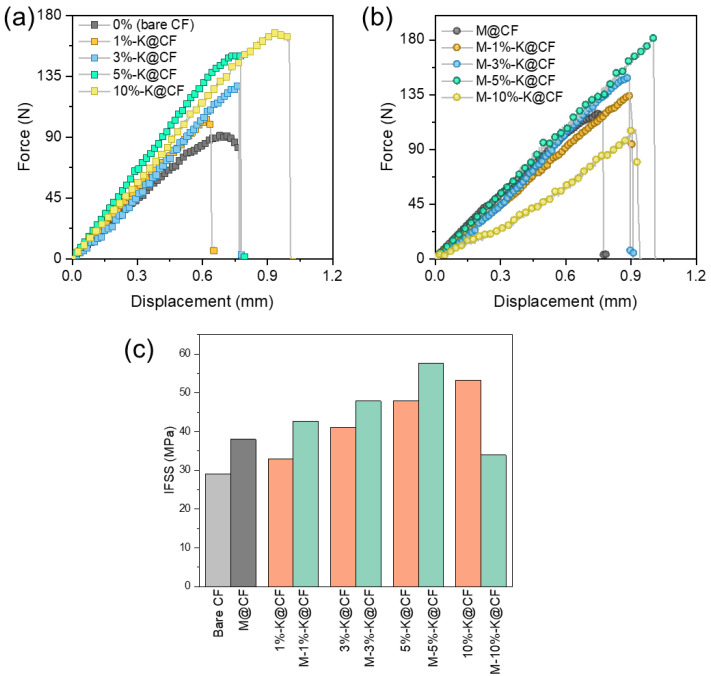
Bundle fiber pull-out tests. Force–displacement curves of (**a**) bare CF with and without KH560, and (**b**) MWCNT-modified CF in (**a**). (**c**) Interfacial shear strength (IFSS), estimated from (**a**,**b**), for bare CF, n-K@CF, M-n-K@CF, and M@CF in resin.

According to previous work [41], the KH560 layer, as the coupling agent, can bridge the CF and the polymer matrix and, hence, enhance the adhesion by forming chemical bonds, while the MWCNTs improve the interface strength by creating physical interlocking. In our case, we have both KH560 and MWCNTs added between the fiber and the matrix; therefore, it is important to elucidate the contributions of each reinforcement, and their collaborative effect on the interfacial properties. We performed fiber yarn pull-out tests on the KH560-modified CFs with and without MWCNTs, respectively, as shown in Figure 5a,b. The bare CF and the MWCNT-modified CFs without KH560 were used as controls. Figure 5a shows the force–displacement curves when the CF yarns with different contents of KH560 were pulled out of the resin matrix, and the IFSS is shown in Figure 5c. Without the interface modification of the CF (bare CF), the IFSS of the CF/resin is about 29 MPa, and it monotonically increases to 53 MPa for the 10%-K@CF/resin. Apparently, the increase in IFSS is relatively faster at a low weight content of KH560 (5%-K@CF), and the increase ratio slows down with further increasing KH560. When incorporating the MWCNTs onto the surface of the n-K@CF, the corresponding force–displacement curves in Figure 5b display a pronounced enhancement in the maximum pull-out force. Its IFSS increases by ~25% compared to that of the KH560-modified CF/resin (n-K@CF). However, a sharp drop in IFSS is found in the M-10%-K@CF/resin, probably due to the agglomeration of the MWCNTs on the fiber surface (Figure 3d). Without the KH560, the IFSS of the MWCNT-modified CF/resin (M@CF/resin) is 30% larger than that of the CF/resin but lower than that of the M-n-K@CF/resin with KH560. These observations suggest that combining the MWCNTs and the KH560 could synergistically improve the interfacial properties, the mechanism by which is discussed next. Essentially, the increase in IFSS is attributed to the increment in adhesion, which could be induced by forming chemical/physical bonding between the CF and the matrix. In our case, the introduction of the coupling agent, the KH560 layer, could assist in the formation of chemical bonds between the CF, the epoxy, and the MWCNTs (Figure S4), which could greatly enhance the adhesion force. Moreover, the MWCNTs could introduce mechanical interlocking at the interface, which could further improve the IFSS. It should be noted that the IFSS strongly depends on the surface roughness, and the relationship between which is complicated. According to the results in Figure 5c and a previous report [11], the IFSS increases with the surface roughness only when the MWCNTs are homogeneously dispersed on the surface. A quantitative study on the effect of surface roughness on the IFSS warrants a separate investigation.

### 3.3. Interfacial Strengthening Mechanism

**Figure 6:**
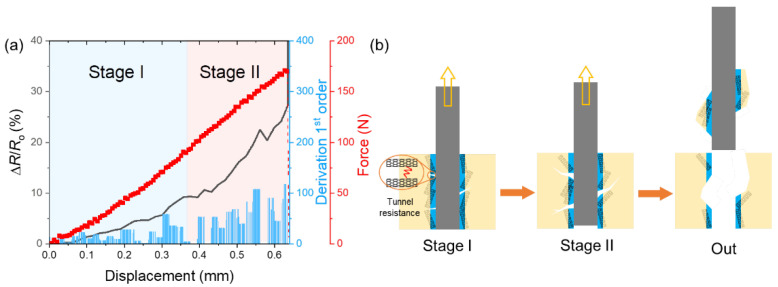
(**a**) The resistance increment ratio, black line, (ΔR/R_o_, ΔR = |R_o_ − R| is the resistance change compared to its initial value R_o_.) as a function of displacement is shown on the left axis, while its 1st-order derivation (blue column) is shown on the right axis. The corresponding load–displacement curve (red line) on the right axis is shown for comparison. The pull-out process is identified as having two stages, the schematic of which is shown in (**b**).

**Figure 7:**
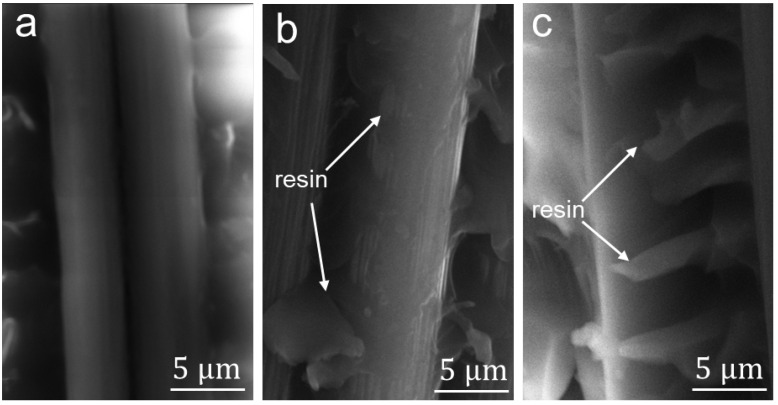
SEM morphology of the fiber surface after a pull-out test: (**a**) CF; (**b**) 5%-K@CF; and (**c**) M-5%-K@CF. The arrows point to the resin left on the CF surfaces.

In light of the sensitivity to electrical resistance of the MWCNTs [11], we utilized the MWCNTs as interfacial damage sensors to in-situ monitor the evolution of the interfacial status in the pull-out tests in order to clarify the origins of increased IFSS in the M-n-K@CF/resin. Figure 6a records the relative resistance (ΔR/R_o_) of the M-5%-K@CF/resin along with its force–displacement curve in the pull-out test, where ΔR = |R_o_ − R| denotes the changes in R compared to its initial value. For the force–displacement curve, the pull-out force increases linearly with increasing displacement, reaching a maximum value of 184 N before it drops to zero. The zero-force suggests a complete debonding between the fiber and the resin that occurs when the fiber is out of the resin. Accordingly, ΔR/R_o_ shows continuous increase with shear force, resulting from reductions in the connection between the MWCNTs that transfers into the tunnel resistance. Despite the increase in ΔR/R_o_ with displacement, the increase in ratio, which is represented by the first-order derivation of ΔR/R_o_, shows a two-stage trend with displacement, as shown in Figure 6a, implying the interfacial status should be different as well. In stage I, the increase in ratio barely changes, while it significantly increases with displacement in stage II. The distinct difference in d(ΔR/R_o_)/d(displacement) suggests that the fiber experiences two different processes before it is totally pulled out of the resin, which is schematically shown in Figure 6b. In stage I, since there is no large drop in resistance, the debonding does not occur and some microcracks in the MWCNT layers should happen, leading to increases in ΔR/R_o_. Indeed, the abundant MWNCTs on the surface usually create the mechanical interlocking to bridge the fiber and the matrix [42], the break between which can produce microcracks along all directions. In contrast, in stage II, the large and fast increase in d(ΔR/R_0_)/d(displacement) implies that numerous debonding between the fiber and the matrix appear and, hence, the cracks can propagate along the interfacial layer direction, leading to detachment between the fiber and the matrix. Given the different affinity of the bare fiber and the resin, their chemical bonding should come from the reaction between the epoxy group of the KH560 and the -NH_2_ group in the resin (Figure S4) [43]. Additionally, the carboxylic groups on the MWCNTs may react with the epoxy (Figure S4) and introduce additional chemical bonds, with the former of KH560 layer being the key factor. The formation of two types of chemical bonding (Figure S4) along with the mechanical interlocking differ from previous work with either MWCNTs or coupling agent-modified CFs [43,44]; the collaborative contributions of the two is expected to yield a larger enhancement in the interfacial properties. To verify this mechanism, we characterized the SEM images of the fiber surface after the pull-out tests in Figure 7. For the M-5%-K@CF, a large amount of residual resin was left on the surface due to the resin fracture, while there was little resin left on the 5%-K@CF and the bare CF. For the latter, its failure modes manifested mainly in the debonding and resin gliding that cost much less energy compared to the resin fracture. For the former MWCNT-modified CFs, the MWCNTs at the interface could interrupt or re-direct the crack propagation and, hence, dissipate more energy. The collaborative contributions of the KH560 and the MWCNTs to the interface strengthening yielded a high IFSS in the modified CF/resin. When compressing the matrix along the transverse direction of the fibers, we found similar results showing the debonding between the fiber and the matrix was the main damage leading to material failure [45].

## 4. Conclusions

In this work, we obtained MWCNT-modified fibers and its composites where the MWCNTs and the coupling agent were introduced using a simple and timesaving two-step route. The MWCNTs were mixed with the coupling agent-coated fibers, where the coupling agent could facilitate the loading of the MWCNTs. The MWCNTs are homogenously distributed on the CF surface, which is confirmed in the SEM images, and its weight content could be directly controlled by the amount of the coupling agent. The MWCNTs and the KH560-modified CF/resin exhibit a larger IFSS than that of the ones without either the coupling agent or the MWCNTs. Its interfacial strengthening mechanism is elucidated through an in-situ monitoring process that is achieved by the MWCNTs with a high electrical conductivity. It reveals that the high interfacial strength is attributed to the chemical bonding formed between the coupling agent and the matrix and the bright effect of the MWCNTs to dissipate energy. In light of its high efficiency and simplicity, this type of MWCNT modification is compatible with industrial applications, and the strong interfacial properties achieved in the CF/resin and the self-monitoring feature offer a lot of potential in structural health monitoring application.

## Data Availability

The original contributions presented in the study are included in the article/Supplementary Materials. Further inquiries can be directed to the corresponding author.

## Supplementary Materials

The following supporting information can be downloaded at https://www.mdpi.com/article/10.3390/nano12213717/s1, Figure S1: SEM images of (a) 1%-K@CF, (b) 3%-K@CF, (c) 5%-K@CF, (d) M@CF, (e) M-1%-K@CF, and (f) M-3%-K@CF; Figure S2: Weight fraction as a function of temperature for CF with and without KH560; Figure S3: SEM images of (a) dispersed MWCNTs and (b) M-5%-K@CF, where the MWCNTs marked with white circles are selected and measured its outer diameter. The corresponding outer diameter distribution (outer diameter vs. probability) is shown in (c); Figure S4: Schematic of chemical bonding formation between CF and matrix.
