# Supplementary material for: Interfacial Strengthening and Self-Monitoring in Carbon Fiber-Reinforced Composites via Carbon Nanotube-Based Damage Sensors
Source: Nanomaterials (Basel). 2022 Oct 22;12(21):3717. doi: 10.3390/nano12213717 (PMC9654919; doi:10.3390/nano12213717)
Supplement: Supplementary file 1 [file nanomaterials-12-03717-s001.zip › nanomaterials-1969145-supplementary.pdf]

Suppurting Information

Interfacial Strengthening and Self-Monitoring in Carbon Fiber-Reinforced Composites via Carbon Nanotube-Based Damage Sensors

Wenlong Hu [1], Zijie Sun [2], Lulu Yang [1], Chaojie Hu [1], Shuzheng Zhang [1], Fangxin Wang [3], Bin Yang [1] and Yu Cang [1,*]

[1] School of Aerospace Engineering and Applied Mechanics, Tongji University, Shanghai 200092, China
[2] Beijing Spacecrafts, China Academy of Space Technology, Beijing 100094, China
[3] College of Civil Science and Engineering, Yangzhou University, Yangzhou 225127, China
* Correspondence: yucang@tongji.edu.cn

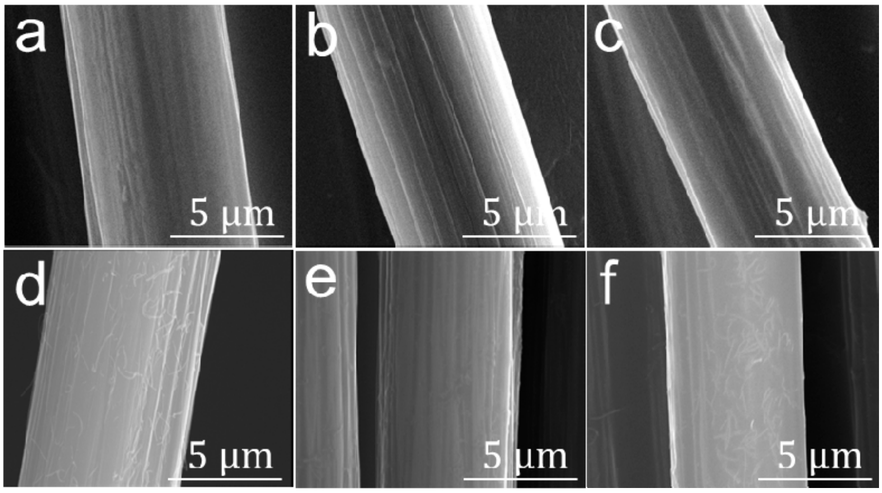

Figure S1. SEM images of (a) 1%-K@CF, (b) 3%-K@CF, (c) 5%-K@CF, (d) M@CF, (e) M-1%-K@CF, and (f) M-3%-K@CF.

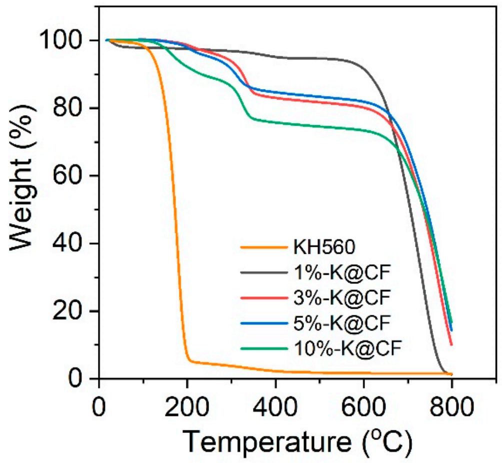

Figure S2. Weight fraction as a function of temperature for CF with and without KH560.

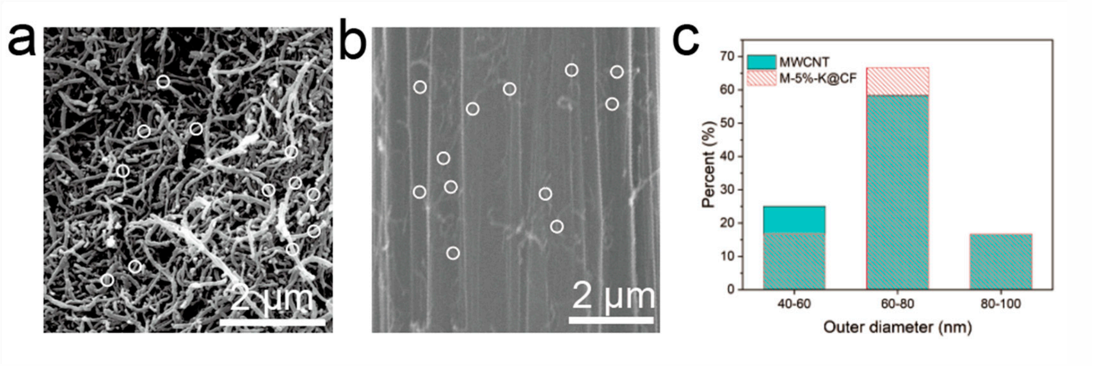

Figure S3. SEM images of (a) dispersed MWCNTs and (b) M-5%-K@CF, where the MWCNTs marked with white circles are selected and measured its outer diameter. The corresponding outer diameter distribution (outer diameter vs. probability) is shown in (c).

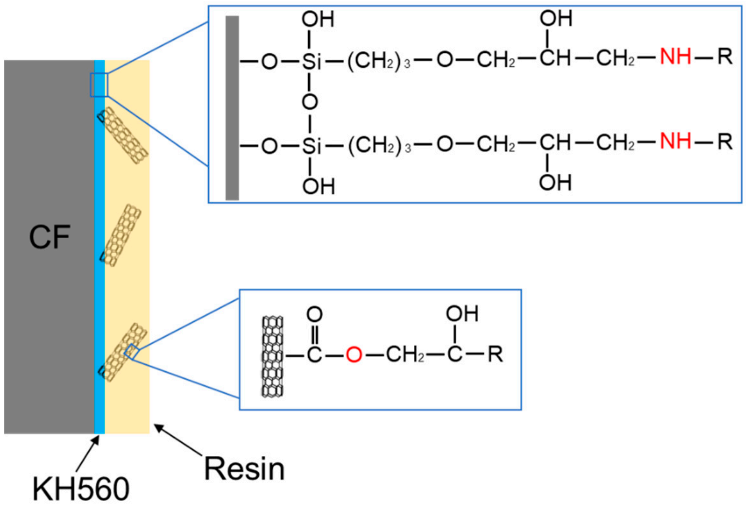

Figure S4. Schematic of chemical bonding formation between CF and matrix.
